# Long Non-coding Antisense RNA TNRC6C-AS1 Is Activated in Papillary Thyroid Cancer and Promotes Cancer Progression by Suppressing TNRC6C Expression

**DOI:** 10.3389/fendo.2018.00360

**Published:** 2018-07-09

**Authors:** Dilidaer Muhanhali, Tianyu Zhai, Jingjing Jiang, Zhilong Ai, Wei Zhu, Yan Ling

**Affiliations:** ^1^Department of Endocrinology and Metabolism, Zhongshan Hospital, Fudan University, Shanghai, China; ^2^Department of General Surgery, Zhongshan Hospital, Fudan University, Shanghai, China

**Keywords:** long non-coding antisense RNA, papillary thyroid cancer, TNRC6C-AS1, TNRC6C, iodine accumulation

## Abstract

**Context:** Evidences have shown the important role of long non-coding antisense RNAs in regulating its cognate sense gene in cancer biology.

**Objective:** Investigate the regulatory role of a long non-coding antisense RNA TNRC6C-AS1 on its sense partner TNRC6C, and their effects on the aggressiveness and iodine-uptake ability of papillary thyroid cancer (PTC).

**Design:** TNRC6C-AS1 was identified as the target long non-coding RNA in PTC by using microarray analysis and computational analysis. *In vitro* gain/loss-of-function experiments were performed to investigate the effects of TNRC6C-AS1 and TNRC6C on proliferation, apoptosis, migration, invasion and iodine-uptake ability of TPC1 cells. Expression levels of TNRC6C-AS1 and TNRC6C of 30 cases of PTC tissues and its adjacent normal thyroid tissues were determined.

**Results:** Downregulation of TNRC6C-AS1 or overexpression of TNRC6C inhibited proliferation, migration and invasion of TPC1 cells, while apoptosis and iodine uptake was promoted in TPC1 cells. Suppression of TNRC6C-AS1 significantly increased the expression of TNRC6C in TPC1 cells. The inhibitory effect of TNRC6C-AS1 knockdown on cell proliferation, migration and invasion was attenuated when the expression of TNRC6C was suppressed simultaneously, indicating TNRC6C is a functional target of TNRC6C-AS1. The expression of TNRC6C-AS1 was significantly higher, while the TNRC6C mRNA and protein were significantly lower in PTC tissues than normal adjacent tissues. There was a significant inverse correlation between TNRC6C-AS1 and TNRC6C mRNA in PTC tissue samples.

**Conclusions:** TNRC6C-AS1 promotes the progression of PTC and inhibits its ability of iodine accumulation by suppressing the expression of TNRC6C. Targeting TNRC6C-AS1 - TNRC6C axis may be a new promising treatment for PTC.

## Introduction

Thyroid cancer is the most common endocrine malignancy worldwide, and its incidence has increased substantially during the past few decades (1–3). Papillary thyroid cancer (PTC) is the most frequent histological type of thyroid cancer, and almost the entire change of the incidence of thyroid cancer has been attributed to an increase in the incidence of PTC (4). As with other types of cancer, thyroid cancer behaves as a complex disease where multiple molecular genetic factors interact with environmental factors (5). Recent studies have focused on the role of long non-coding RNAs (lncRNAs) in cancer pathogenesis. Several lncRNAs were reported to be dysregulated in PTC and participate in the carcinogenesis and progression of PTC (6–9). Natural antisense transcripts (NATs), defined as transcribed RNA products complementary to their endogenous sense or protein coding transcripts, frequently do not encode proteins. It was reported that up to 72% of all genome-mapped transcriptional units can produce transcripts from both strands (10). Recent evidence indicates that NATs are frequently functional and carry out a wide variety of biological roles through diverse transcriptional and post-transcriptional gene regulatory mechanisms (11). Many studies have shown the important role of long non-coding antisense RNAs in regulating its cognate sense gene in cancer biology (12, 13).

In the current study, we focused on the regulatory effects of one particular long non-coding antisense RNA uc002juh.2 (TNRC6C antisense transcript 1, TNRC6C-AS1) in PTC. Microarray analysis identified marked and consistent upregulation of TNRC6C-AS1 in human PTC tissues relative to adjacent normal tissues. We found that the TNRC6C-AS1 is an antisense oligonucleotide RNA transcribed form the reverse strand of Homo sapiens trinucleotide repeat containing 6C (TNRC6C), a protein-coding gene. The TNRC6C protein belongs to the GW182 family, which has three paralogs in mammals (14). The human members of this protein family are designated as trinucleotide repeat containing (TNRC)-6A, 6B, and 6C (14). All three TNRC6 proteins which can interact with members of the Argonaute subfamily, are essential components of mRNA-processing bodies (P-bodies) and are required for miRNA-mediated mRNA repression in mammalian cells (14–17).

A few studies have investigated the role of TNRC6 proteins in cancers. Kim et al. found that frameshift mutations in TNRC6A were common in gastric cancers and colorectal cancers with high microsatellite instability and loss of TNRC6A expression was observed in over 50% of these cancers (18). Je et al. also found that TNRC6A was not expressed in 20% non-small cell lung cancers, while TNRC6A was well expressed in normal bronchial epithelial cells (19). These data suggests that loss of TNRC6A expression may be a feature of several cancers. In another study, TNRC6C was demonstrated to be the downstream regulated gene of GAEC1 oncogene in esophageal squamous cell carcinoma (20). When GAEC1 expression was suppressed, there was significant upregulation of TNRC6C expression in esophageal carcinoma cell line (20). The effect of TNRC6C-AS1 or TNRC6 proteins on thyroid cancer remains unknown. Based on the important role of TNRC6 proteins in miRNA-dependent post-transcriptional silencing pathway and the fact that loss of TNRC6 protein expression in cancers, the regulatory role of TNRC6C-AS1 and TNRC6C may be important in the development and progression of PTC.

In this study, we demonstrated the effect of TNRC6C-AS1 and TNRC6C on proliferation, apoptosis, migration and invasion of PTC cells. The regulatory role of TNRC6C-AS1 on TNRC6C in PTC cells was investigated. The effect of TNRC6C-AS1 and TNRC6C on the ability of iodine-uptake of PTC cells was also explored. Although the 10-year survival rates in patients with differentiated thyroid cancer are considered excellent (21), there is a small group of patients that may have poor prognosis with a 10-year survival rate as low as 20–50%, such as in the cases with distant metastasis (22, 23). A major reason for these patients' poor outcome is their resistance to radioiodine therapy due to the loss of iodine accumulation ability of thyroid cancer cells. In fact, 7–23% of thyroid cancer cases develop into distant metastases (24) and from those around two-thirds become iodine refractory during follow-up which results in a 10-year survival lower than 10% (23). Effective radioiodide therapy of metastatic thyroid cancers requires uptake of radioiodide via the sodium/iodide symporter (NIS), and optimally, its incorporation into tyrosine residues of thyroglobulin to increase the residence time of the isotope. We investigated if the TNRC6C-AS1–TNRC6C axis regulate the expression of genes required for thyroid hormone biosynthesis, including NIS, thyroid stimulating hormone receptor (TSHR), thyroid peroxidase (TPO), and Pendrin. We hypothesized that targeting TNRC6C-AS1–TNRC6C axis can inhibit PTC progression, restore the expression of these thyroid differentiation genes, and increase iodine accumulation in PTC cells.

## Materials and methods

### Patients and samples

This study recruited 33 PTC patients who were admitted to Zhongshan Hospital, Fudan University, Shanghai, China between July 2016 and July 2017. All patients were pathologically confirmed as PTC. Surgical specimens including PTC cancer tissues and adjacent noncancerous tissues were obtained during thyroidectomy. All tissue samples were stored in liquid nitrogen. Among these paired tissue samples, three samples were used for lncRNA microarray analysis, and the other 30 samples were used to determine the expression levels of TNRC6C-AS1 and TNRC6C. This study was approved by the Ethics Committee of Zhongshan Hospital, Fudan University, Shanghai, China. Written informed consents were obtained from all enrolled patients.

### lncRNA microarray

Three PTC tissues and three paired normal tissues were sent to Kangchen Biological Services (Shanghai, China) for microarray hybridization and data analysis. Total RNA were extracted from six tissue samples and hybridized to the gene chip using Human LncRNA Array v2.0 (Arraystar Inc., Rockville, MD, USA) based on the manufacturer's instructions. The arrays were scanned by Agilent Scanner (G2505B; Agilent Technologies Inc., Santa Clara, CA, USA) and acquired raw images were analyzed using Agilent Feature Extraction software (version 11.0.1.1). The GeneSpring GX v11.5.1 software package offered subsequent data processing. Volcano Plot filtering was carried out and the threshold was set as fold change >2 and *P* < 0.05 between cancer and noncancerous tissues. Hierarchical clustering was carried out to show the distinguishable lncRNAs expression pattern between PTC tissues and adjacent normal tissues.

### RNA isolation and real-time qPCR analysis

Total RNA was extracted from cells or tissues using the TRIzol Reagent (Takara, Kusatsu, Japan) following the manufacturer's instructions, and 1 ug of total RNA was used for synthesizing cDNA by Reverse Transcription Kit (Takara, Kusatsu, Japan). Real-time qPCR was performed to assess the expression level of each gene using the SYBR Green PCR Kit (Takara, Kusatsu, Japan) in the ABI7500/Viia7 real-time PCR detection system (Applied Biosystems, Foster City, CA, USA). GAPDH or β-actin was used to normalize the expression levels of target genes. The primers used in this study were listed in Supplementary Table S1.

### Western blot assay

Total proteins were extracted from cells or tissues using a radio immunoprecipitation assay (RIPA) buffer (Beyotime, Shanghai, China) containing proteinase inhibitors. A bicinchoninic acid (BCA) assay (Thermo, Rockford, IL, USA) was performed to measure protein concentrations. 30 μg of extracted proteins per well was loaded onto 8–12% SDS-PAGE gel (Beyotime, Shanghai, China) for electrophoresis and transferred to a polyvinylidene fluoride (PVDF) membrane (Millipore, Billerica, MA). The PVDF membranes were blocked with 5% milk solution for 2 h and then incubated with primary antibody diluted in 5% bovine serum albumin (Sigma, St Louis, MO, USA) overnight at 4°C. After that, membranes were washed in 0.1% PBS/Tween-20 (PBST) and probed with secondary antibody for 1.5 h at room temperature. After being washed 3 times in PBST again, the membranes were visualized using the ChemiDoc XRS System (BioRad, Hercules, CA) by enhanced chemiluminescence (ECL) detecting kit (Thermo, Rockford, IL, USA). Antibodies applied in this study were anti-TNRC6C antibody (Santa Cruz, sc-244474), anti-TSHR antibody (Proteintech, 14450-1-AP), anti-TPO antibody (Affinity, DF8279), anti-Pendrin antibody (Santa Cruz, sc-50346), anti-NIS antibody (Affinity, DF2242), anti-GAPDH antibody (Bioworld, AP0063) and anti-β-actin antibody (Proteintech, 60008-1-IG).

### Cell culture and transfection

The expression of TNRC6C-AS1 was first examined in 3 human PTC-derived cell lines (TPC1, BCPAP, and K1) and a normal thyroid epithelial cell line (Nthy-ori3-1). All these cell lines were obtained from the Cell Bank of Chinese Academy of Sciences (Shanghai, China) and maintained in humidified atmosphere of 37°C and 5% CO_2_. Cells were grown in DMEM (HyClone, Logan, UT, USA) supplemented with 10% fetal bovine serum, 100 U/ml penicillin and 100 mg/ml streptomycin. For overexpression of TNRC6C or TNRC6C-AS1, the vectors expressing TNRC6C or TNRC6C-AS1 were prepared by amplifying full length of complementary cDNA encoding TNRC6C or TNRC6C-AS1 and the amplified fragments were then cloned into pcDNA3.1 vector (Invitrogen, Carlsbad, CA, USA). TPC1 cells were transfected with the TNRC6C or TNRC6C-AS1 overexpression plasmid using Lipofectamine 2000 (Invitrogen, Carlsbad, CA, USA) transfection reagent according to the protocols. The efficiency of overexpression plasmids were measured by real-time qPCR 48 h post-transfection. For downregulation of TNRC6C-AS1 or TNRC6C, three different short interfering RNA (siRNAs) specifically against TNRC6C-AS1 or TNRC6C were designed and synthesized by Genepharma Company (Shanghai, China) and transiently transfected into TPC1 cells using Lipofectamine 2000 (Invitrogen, Carlsbad, CA, USA) according to the manufacturer's instructions. Real-time qPCR was performed to measure the transfection efficiency of different siRNAs. Cells transfected with siRNA-control or empty vector were used as negative controls. All siRNA sequences were listed in Supplementary Table S1.

### Cell proliferation assay

Cell proliferation was determined using cell counting kit-8 (CCK-8) (Dojindo, Kumamoto, Japan).100 ul of TPC1 cell suspension were seeded into 96-well plates at a concentration of 1 × 10^5^ cells/well and transfected for 48 h. Afterward, 10 μl CCK-8 solution was added to each well and incubated at 37°C for 4 h. The absorbance at 450 nm was measured using a Microplate Reader (RT6000, Rayto, Shenzhen, China).

### Flow cytometry assay

Cell apoptosis was measured using Cell Cycle and Apoptosis Analysis Kit (Beyotime, Shanghai, China). After transfection for 48 h, TPC1 cells were harvested using trypsin and washed twice with phosphate-buffered saline (PBS). Then, the cells were suspended by 500 ul binding buffer with 5 ul Annexin V-FITC and 5 ul propidium iodide, and incubated at 25°C for 15 min in the dark. After that, the cells were analyzed on a FACS Calibur flow cytometer (BD Biosciences, Franklin Lakes, NJ, USA). The percentage of apoptosis cells was the proportion of the early and late apoptosis cells.

### Wound-healing assay

To evaluate cell migration, 2 × 10^5^ cells were seeded into each well of 6-well plates and cultured until the cells reached 70–90% confluence. After transfection for 48 h, scratch wounds were created by a pipette tip. The streaked cells were then washed with PBS 3 times and cultured in serum-free medium. Images of the same fields were taken at 0 and 24 h. ImageJ software (National Institutes of Health, Bethesda, MD, USA) was applied to measure the relative migration area.

### Transwell invasion assay

Transwell invasion assay was carried out using 8.0 um Transwell chambers (Corning, NY, USA). Transfected cells were suspended in 200 μl serum-free medium and placed into the upper chamber of a transwell chamber pre-coated with Matrigel Matrix (BD Biosciences, Franklin Lakes, NJ, USA). The lower chamber was filled with 600 μl medium with 10% FBS. After incubation at 37°C for 24 h, the cells that traversed the filter were fixed with 4% paraformaldehyde for 30 min, stained with 0.1% crystal violet for 20 min and photographed under a fluorescence microscope (Olympus, Tokyo, Japan) at 3 different fields per filter.

### Luciferase reporter assay

The 3′UTR segments of human TNRC6C contains the putative binding site for TNRC6C-AS1 were amplified by PCR and was inserted between the restrictive sites Xho I and Not I of the psiCHECKTM-2 Vector (Promega, Madison, WI, USA). The putative binding site of TNRC6C-AS1 in TNRC6C was mutated by using a QuikChange Site-Directed Mutagenesis kit (Agilent, USA) to synthetize pMIR-TNRC6C 3′UTR-mut. TPC1 cells were cultured in a 96-well plate until they reached 70% confluence. Then, the cells were co-transfected with 100 ng of luciferase construct pMIR-TNRC6C 3′UTR or pMIR-TNRC6C 3′UTR-mut, 10 ng of Renilla luciferase vector and 100 nM TNRC6C-AS1 overexpression plasmid or negative control using Lipofectamine 2000 (Invitrogen, Carlsbad, CA, USA). After co-transfecting for 48 h, TPC1 cells were washed by PBS and lysed by Passive Lysis Buffer (PLB) for 15 min. The relative luciferase activity was normalized to Renilla luciferase activity using Dual-Luciferase Reporter Assay System (Promega, Madison, USA). The sequence of 3′UTR segments of human TNRC6C was listed in Supplemental Table S1.

### Iodine uptake assay

The iodine uptake of TPC1 cell was measured by using Sodium Thiosulfate (Na_2_S_2_O_3_) Standard Titration. After transfecteing for 48 h, TPC1 cells were treated with medium adding potassium iodade (KIO_3_) solution at a concentration of 5,000 μg/L. After cultured for different time (0, 6, 12, 24, and 48 h), cells were ruptured and the supernatant was collected. Afterwards, 4 ml of supernatant was placed in a 250 ml iodine flask containing 50 ml of double-distilled water, 1 ml of sulfuric acid (H_2_SO_4_) solution and 5 ml potassium iodide (KI) solution. The flask was covered with a stopper and put in dark for 5 min. The sample solution was titrated by Na_2_S_2_O_3_ standard titrant (0.1 mol/L) until the solution became pale yellow. Then 2 ml of starch indicator was added into the sample solution. The sample solution was titrated with constant stirring until the blue color disappeared. The burette mark was recorded and the iodine concentration in the supernatant was calculated using the following equation:

$$\begin{array}{l} {\text{C}}_{\left(\text{KI}{\text{O}}_{3}\right)}=\frac{{\text{C}}_{\left(\text{N}{\text{a}}_{2}{\text{S}}_{2}{\text{O}}_{3}\right)}*{\text{V}}_{\left(\text{N}{\text{a}}_{2}{\text{S}}_{2}{\text{O}}_{3}\right)}}{6*{\text{V}}_{\left(\text{KI}{\text{O}}_{3}\right)}}\\ {\text{V}}_{\left(\text{KI}{\text{O}}_{3}\right)}=4\text{ml} \end{array}$$

The chemical equations involved are as follows:

$$\begin{array}{r} \text{KI}{\text{O}}_{3}+5\text{KI}+3{\text{H}}_{2}\text{S}{\text{O}}_{4}=3{\text{K}}_{2}\text{S}{\text{O}}_{4}+3{\text{I}}_{2}+3{\text{H}}_{2}\text{O}\\ {\text{I}}_{2}+2\text{N}{\text{a}}_{2}{\text{S}}_{2}{\text{O}}_{3}=2\text{NaI}+\text{N}{\text{a}}_{2}{\text{S}}_{4}{\text{O}}_{6} \end{array}$$

### Statistical analysis

Data were presented as means ± SD. Paired *t*-test was used to compare expression levels of TNRC6C-AS1 and TNRC6C in PTC tissues and adjacent noncancerous tissues. Pearson correlation analysis was performed to determine the relationship between the expression of TNRC6C-AS1 and TNRC6C mRNA in PTC tissues. Independent *t*-test was used for the comparison between cell groups. *P* < 0.05 was considered statistically significant. SPSS Statistics version 20 (IBM, Chicago, IL, USA) was used for statistical analysis.

## Results

### Microarray analysis revealed that lncRNA TNRC6C-AS1 is upregulated in PTC

**Figure 1:**
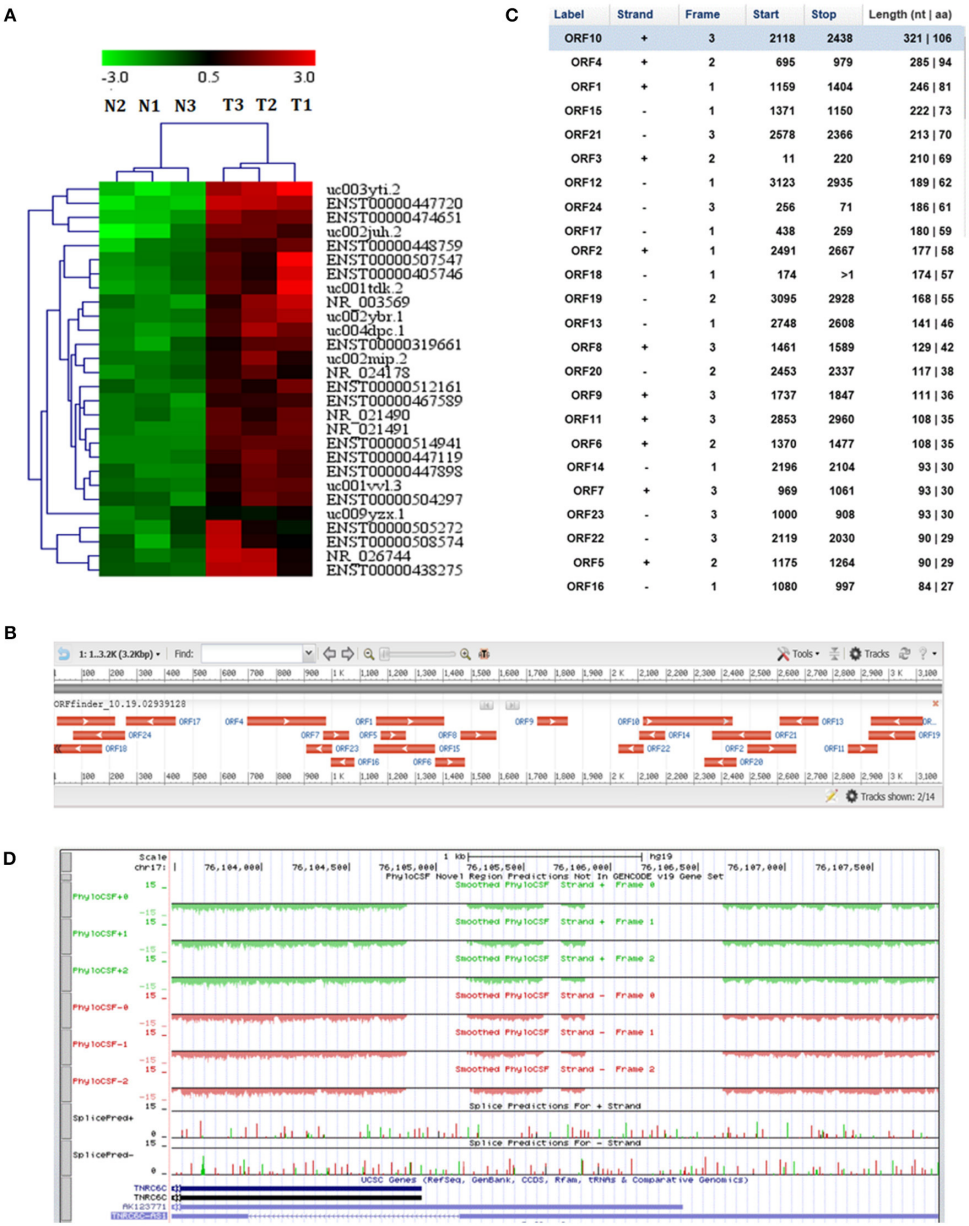
LncRNA TNRC6C-AS1 (uc002juh.2) identified from microarray analysis. **(A)** Hierarchical clustering analysis on the most significantly upregulated cancer-related lncRNAs in PTC resulted from microarray assay (fold change ≥ 4.0, *P* < 0.05). T1, T2, T3 represented for 3 PTC tissues, and N1, N2, N3 represented for their paired adjacent normal tissues. Red indicates a high relative expression and green indicates a low relative expression. **(B)** Open reading frame (ORF) prediction of TNRC6C-AS1 sequence by ORF Finder showed that no continues ORF was found. **(C)** ORF Finder showed that the longest ORF of TNRC6C-AS1 has 106 amino acid and the blast results showed that these ORFs have no homologous proteins. **(D)** The codon substitution frequency scores (CSF) of TNRC6C-AS1 by PhyloCSF was lower than 0, indicating that it is not a conserved sequence.

Using microarray analysis, we examined the lncRNA expression profile in 3 paired human PTC and adjacent noncancerous tissue samples. In order to identify differently expressed lncRNAs, we set fold change >2 and *P* < 0.05 between cancer and noncancerous tissues as the criteria. Microarray analysis revealed that 232 lncRNAs were significantly differentially expressed in PTC tissues compared with their adjacent noncancerous tissues. Among them, 41 were up-regulated and 191 were down-regulated. The most significantly up-regulated lncRNAs with a fold change ≥4.0 and *P* < 0.05 in microarray data were selected for hierarchical clustering analysis (Figure 1A). Through further literature search of distinguishable lncRNAs and adjacent coding genes, we found that antisense lncRNA uc002juh.2 (TNRC6C-AS1) and its cognate sense gene TNRC6C were most likely to be involved in tumorigenesis. Therefore, we selected lncRNA TNRC6C-AS1 for further study.

In order to understand the protein coding potential of TNRC6C-AS1, we analyzed the sequence of TNRC6C-AS1 using ORF Finder. No continuous ORF was found as showed in Figure 1B. The longest ORF of TNRC6C-AS1 has 106 amino acids and the blast results showed that these ORFs have no homologous proteins (Figure 1C). In addition, further analysis was performed using PhyloCSF in UCSC. The results showed that the codon substitution frequency score (CSF) of lncRNA TNRC6C-AS1 was lower than 0, indicating that it is not a conserved sequence (Figure 1D). These evidence indicates that TNRC6C-AS1 has no protein coding potential and meets the characteristic of a lncRNA.

### Downregulation of TNRC6C-AS1 suppresses the proliferation and migration of TPC1 cells

**Figure 2:**
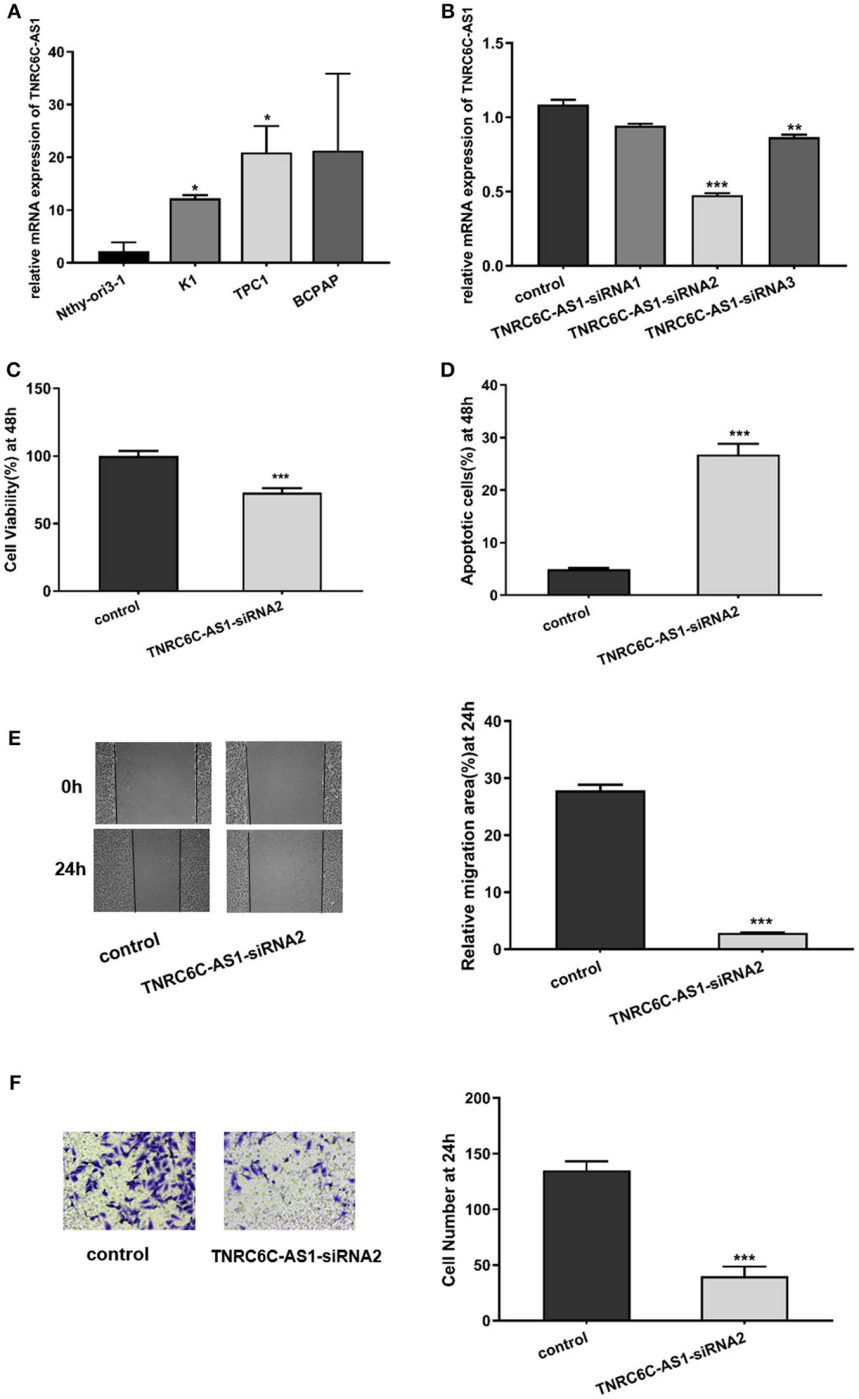
Downregulation of TNRC6C-AS1 suppresses proliferation and migration of TPC1 cells. **(A)** The relative mRNA level of TNRC6C-AS1 in PTC-derived cell lines (TPC1, BCPAP, and K1) and normal thyroid epithelial cell line (Nthy-ori3-1).TNRC6C-AS1 was significantly up-regulated in TPC1 and K1 cells relative to Nthy-ori3-1, and expression level of TNRC6C-AS1 was near 2-fold higher in TPC1 cells compared to K1 cells. **(B)** The relative mRNA level of TNRC6C-AS1 in TPC1 cells after transfected with three different siRNAs targeting TNRC6C-AS1 for 48 h. TNRC6C-AS1 was efficiently silenced by TNRC6C-AS1-siRNA2 compared to other two siRNAs. **(C)** Proliferation assay by CCK-8 in TPC1 cells. Downregulation of TNRC6C-AS1 by transfecting of siRNA-TNRC6C-AS1 for 48 h in TPC1 cells led to a 30% decrease of cell viability (OD 450 nm) compared to control cells. **(D)** Apoptosis assay by flow cytometry in TPC1 cells. Downregulation of TNRC6C-AS1 by transfecting of siRNA-TNRC6C-AS1 for 48 h caused a near 6-fold increase of apoptosis of TPC1 cells compared to the control cells. **(E)** Wound-healing assay in TPC1 cells. Significantly more cells migrated in control cells than in siRNA-TNRC6C-AS1 transfected cells at 24 h [representative images (left) and quantitative analysis (right)]. **(F)** Transwell assay in TPC1 cells. The siRNA-TNRC6C-AS1 transfected TPC1 cells showed significant reduction in cell invasion compared to control cells at 24 h [representative images (left) and quantitative analysis (right)]. *P < 0.05; **P < 0.01; ***P < 0.001.

Expression level of TNRC6C-AS1 was determined in human PTC-derived cell lines (TPC1, BCPAP, and K1) and the normal thyroid epithelial cell line (Nthy-ori3-1). The results showed that TNRC6C-AS1 was upregulated in TPC1 and K1 cells relative to Nthy-ori3-1 cell (Figure 2A). Furthermore, the expression level of TNRC6C-AS1 was nearly 2-fold higher in TPC1 cells compared to the level in K1 cells (Figure 2A). Therefore, we used TPC1 cells for further experiments.

To explore the functions of TNRC6C-AS1 in TPC1 cells, we knocked down TNRC6C-AS1 via transfecting three siRNAs specifically targeting TNRC6C-AS1. Among them, TNRC6C-AS1-siRNA2 efficiently knocked down TNRC6C-AS1 expression (Figure 2B). CCK-8 assay was performed for the examination of cell proliferation. The results showed that depletion of TNRC6C-AS1 significantly suppressed TPC1 cell proliferation at 48 h after transfection (Figure 2C). In addition, apoptosis analysis by flow cytometry revealed that downregulation of TNRC6C-AS1 promoted TPC1 cell apoptosis at 48 h after transfection (Figure 2D). To investigate the effect of TNRC6C-AS1 on migratory and invasive abilities of TPC1 cells, wound-healing assay and transwell assay were performed. The wound-healing assay revealed that downregulation of TNRC6C-AS1 slowed down the closing rate of scratched wounds after 24 h (Figure 2E). Besides, the transwell assay showed that TNRC6C-AS1 downregulation suppressed cell invasion ability at 24 h (Figure 2F). In summary, we considered that downregulation of TNRC6C-AS1 suppressed the aggressiveness of TPC1 cells.

### TNRC6C-AS1 regulates the expression of TNRC6C

**Figure 3:**
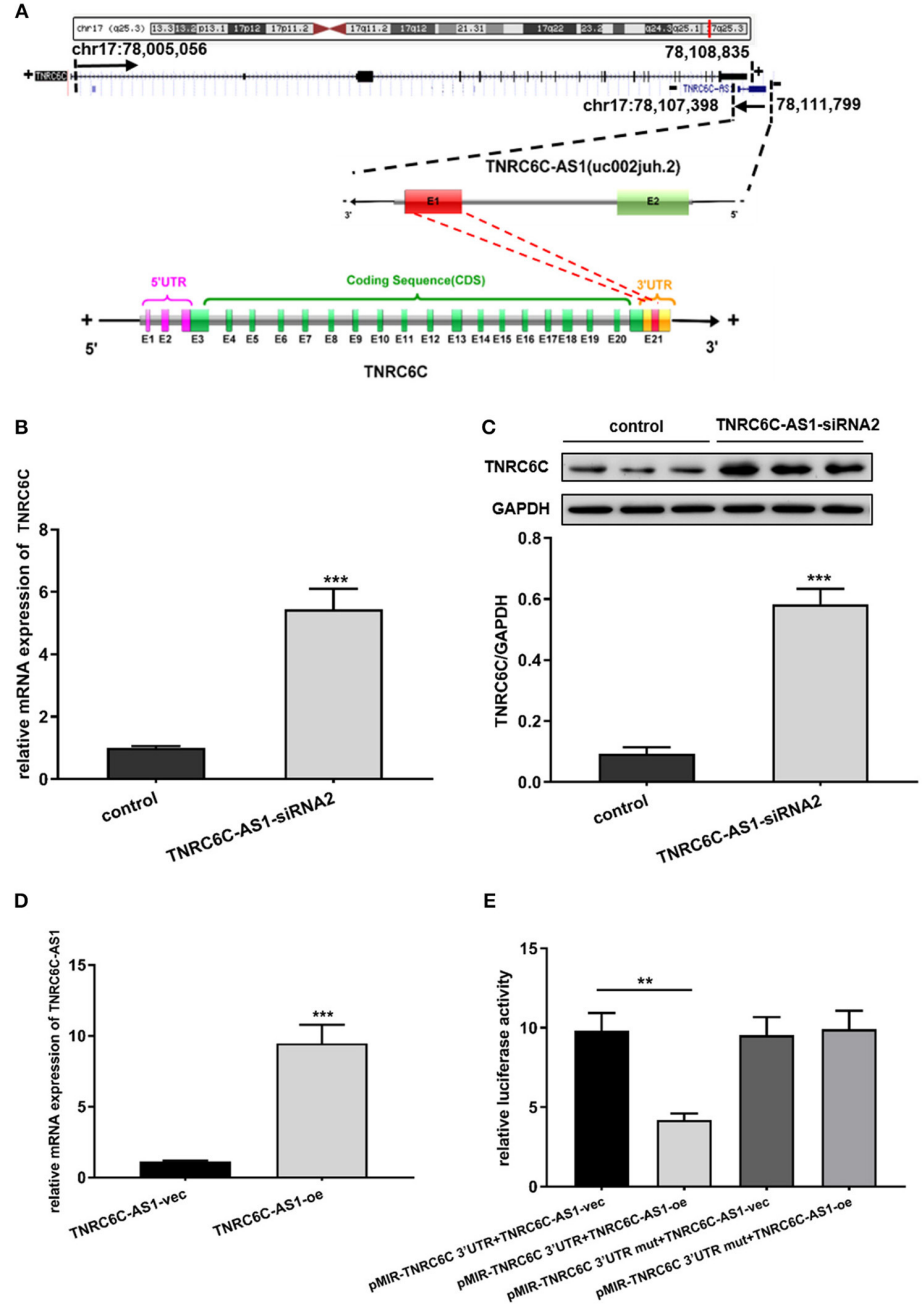
TNRC6C-AS1 regulates TNRC6C expression at both the mRNA and protein levels. **(A)** Schematic outlining the genomic organization of TNRC6C-AS1 and TNRC6C. Upper chart of this panel shows genome organization of the TNRC6C gene at locus 17q25.3. It shows the start and end positions of TNRC6C as indicated on the UCSC site (Genome Bioinformatics). Black arrows indicate transcription direction. Lower chart of this panel is a schematic representation of TNRC6C-AS1 (RefSeq NR_040071) and TNRC6C mRNA (RefSeq NM_001142640). The schema is not drawn to scale. Blocks with colors (green and red) represent exons. Yellow blocks represents 3′UTR and pink block represent 5′UTR. Block filed with gray color represents introns. Red block in TNRC6C-AS1 and TNRC6C represents their overlapping region. “E” represents exon and the following number represents serial number. “+” and “–” at both sides of the strands represents the positive strand and the negative strand respectively. **(B)** Downregulation of TNRC6C-AS1 by specific siRNA in TPC1 cells led to a more than 4-fold increase of TNRC6C mRNA expression compared to siRNA-control transfected cells. **(C)** Downregulation of TNRC6C-AS1 caused a near 6-fold increase of TNRC6C protein levels in TPC1 cells compared to control cells. **(D)** Real-time qPCR showed that mRNA levels of TNRC6C-AS1 was near 9-fold higher in TPC1 cells transfected TNRC6C-AS1 overexpression plasmid relative to empty vector transfected cells. **(E)** Luciferase reporter assay showed that overexpression of TNRC6C-AS1 led to a 50% decrease of luciferase activity of pMIR-TNRC6C 3′UTR compared to control cells. But luciferase activity of pMIR-TNRC6C 3′UTR-mut had no significant change after co-transfecting with TNRC6C-AS1 overexpression plasmid compared to control cells. oe, overexpression plasmid; vec, empty vector; **P < 0.01; ***P < 0.001.

From the UCSC genome browser, uc002juh.2 (RefSeq NR_040071) was identified as a single antisense RNA located at chromosomal band 17q25.3, consist of 4402 nucleotides. uc002juh.2 was named as TNRC6C-AS1, which shared an overlapping region with the 3′UTR region of TNRC6C (RefSeq NM_001142640) (Figure 3A).

Because of its nearby genomic location to TNRC6C, we transiently down-regulated TNRC6C-AS1 in TPC1 cells to identify whether there is a direct link between the expression of TNRC6C-AS1 and TNRC6C. We found that downregulation of TNRC6C-AS1 restored the expression of TNRC6C at both the mRNA and protein levels in TPC1 cells (Figures 3B,C).

We overexpressed TNRC6C-AS1 in TPC1 cells via transfecting TNRC6C-AS1 overexpression plasmid. Relative to TPC1 cells transfected with empty vector, the mRNA level of TNRC6C-AS1 was nearly 9-fold higher in TPC1 cells treated with TNRC6C-AS1 overexpression plasmid(Figure 3D). Luciferase reporter assay was performed to determine whether TNRC6C-AS1 could regulate TNRC6C expression through their overlapping region in 3'UTR. The result showed that overexpression of TNRC6C-AS1 led to a significant decrease of luciferase activity of pMIR-TNRC6C 3′UTR. But luciferase activity of pMIR-TNRC6C 3′UTR-mut had no significant change after co-transfecting with TNRC6C-AS1 overexpression plasmid (Figure 3E).

### TNRC6C is a functional target of TNRC6C-AS1

**Figure 4:**
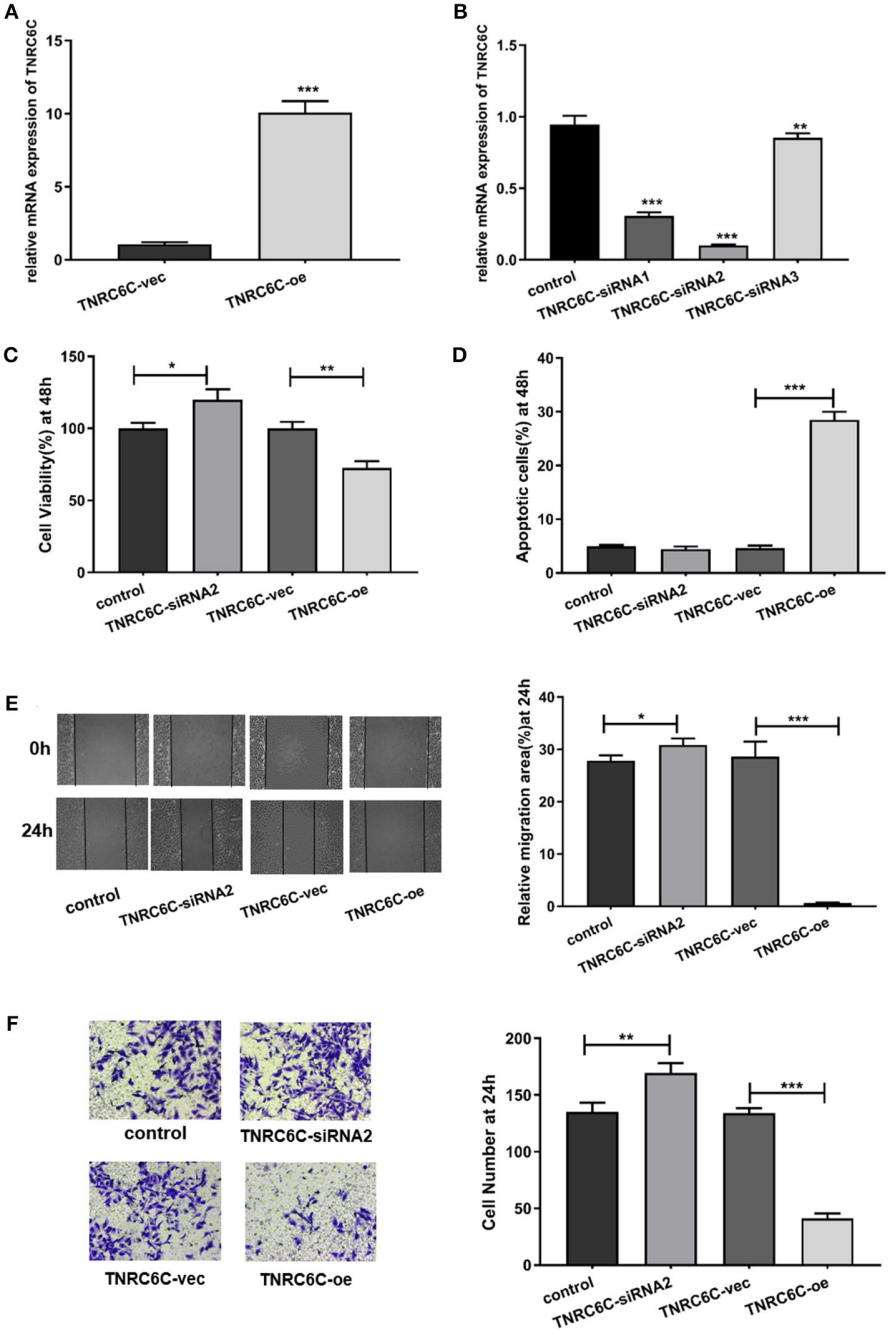
Overexpression of TNRC6C in TPC1 cells leads to decreased proliferation and migration. **(A)** Real-time qPCR showed that mRNA levels of TNRC6C was near 10-fold higher in TPC1 cells transfected TNRC6C overexpression plasmid compared to empty vector transfected cells. **(B)** Real-time qPCR showed that TNRC6C was efficiently silenced by TNRC6C-siRNA2 compared to other two siRNAs. **(C)** Proliferation assay by CCK-8 in TPC1 cells. Overexpression of TNRC6C in TPC1 cells led to a 30% reduction of cell viability (OD 450 nm) compared to the control cells. Downregulation of TNRC6C by specific siRNA in TPC1 cells led to a 20% increase of cell viability compared to the control cells. **(D)** Apoptosis assay by flow cytometry in TPC1 cells. Overexpression of TNRC6C in TPC1 cells caused a near 6-fold increase of apoptosis compared to the control cells. Downregulation of TNRC6C in TPC1 cells didn't cause a significant change of apoptosis compared to the control cells. **(E)** Wound-healing assay in TPC1 cells. Overexpression of TNRC6C in TPC1 cells limited the migration of cells compared to control cells at 24 and 48 h. Significantly more cells migrated in siRNA-TNRC6C transfected cells than control cells at 24 h [representative images (left) and quantitative analysis (right)]. **(F)** Transwell assay in TPC1 cells. TNRC6C over-expressed TPC1 cells showed significant reduction in cell invasion compared to control cells at 24 h. Downregulation of TNRC6C caused significant increase of cell invasion compared to control cells at 24 h [representative images (left) and quantitative analysis (right)]. vec, empty vector; oe, overexpression vector; *P < 0.05; **P < 0.01; ***P < 0.001.

After demonstrating the regulatory effect of TNRC6C-AS1 on TNRC6C, we further investigated the effects of TNRC6C on behaviors of TPC1 cells. We overexpressed TNRC6C in TPC1 cells via transfecting TNRC6C overexpression plasmid. The mRNA level of TNRC6C was nearly 10-fold higher in TPC1 cells transfected with TNRC6C overexpression plasmid compared to TPC1 cells transfected with empty vector (Figure 4A). Overexpression of TNRC6C in TPC1 cells significantly inhibited cell proliferation and promoted cell apoptosis (Figures 4C,D). Consistently, overexpression of TNRC6C caused substantial decline in cell migration and invasion abilities (Figures 4E,F).

We also observed the functions of TNRC6C by down-regulating its expression in TPC1 cells. The knockdown efficiency was confirmed using real-time qPCR and TNRC6C-siRNA2 was selected for the further experiments (Figure 4B). The results showed that downregulation of TNRC6C expression promoted the aggressiveness of TPC1 cells (Figures 4C–F).

**Figure 5:**
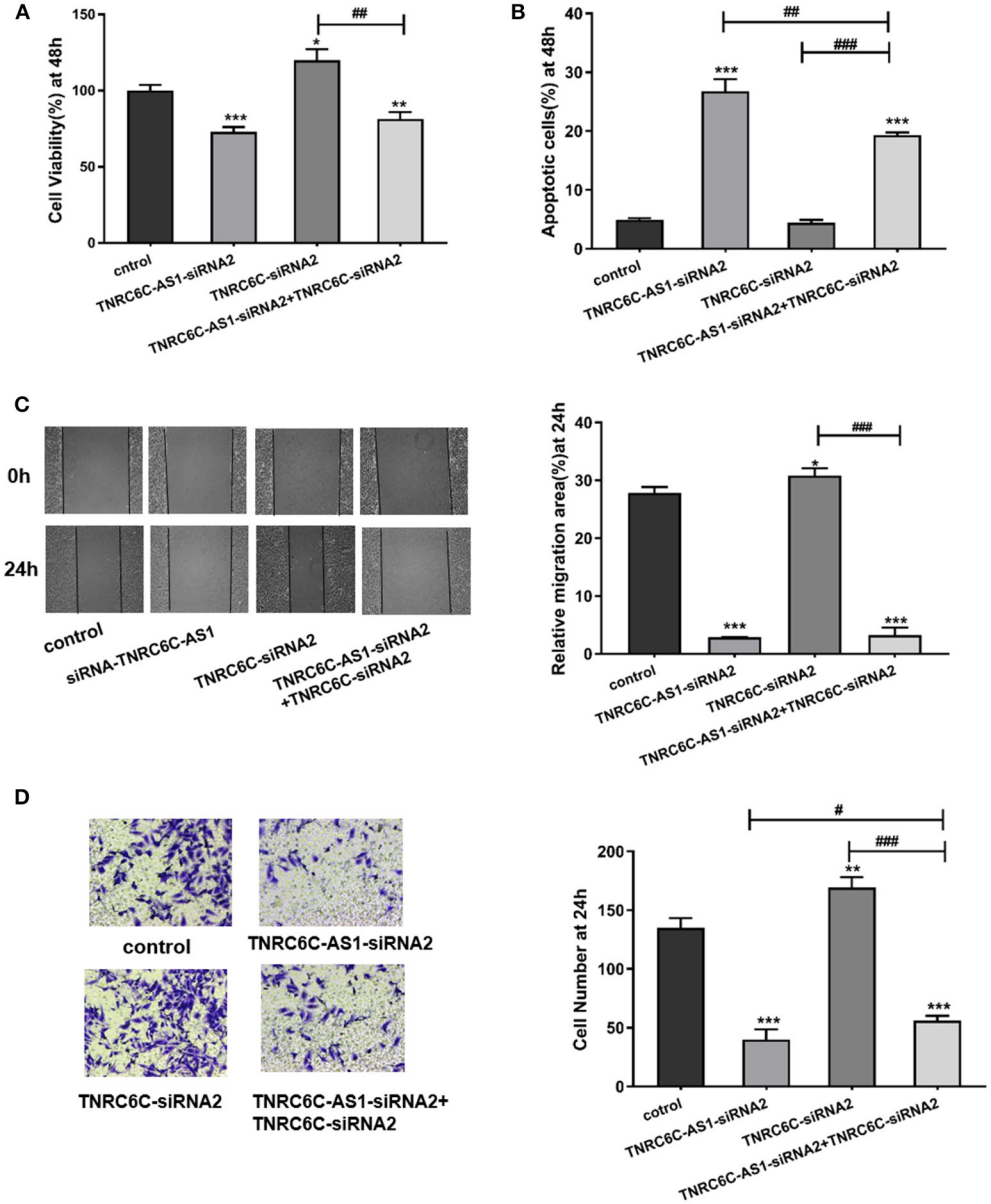
TNRC6C is a functional target of TNRC6C-AS1. **(A)** Proliferation assay by CCK-8 in TPC1 cells. The inhibitory effect of TNRC6C-AS1 knockdown on cell proliferation was attenuated when the expression of TNRC6C was suppressed by co-transfecting with TNRC6C-siRNA2. The promotive effect of TNRC6C knockdown on cell proliferation was reduced by co-transfecting with TNRC6C-AS1-siRNA2. **(B)** Apoptosis assay by flow cytometry in TPC1 cells. The promotive effect of TNRC6C-AS1 knockdown on cell apoptosis was decreased by co-transfecting with TNRC6C-siRNA2. The inhibotory effect of TNRC6C knockdown on cell apoptosis was reduced by co-transfecting with TNRC6C-AS1-siRNA2. **(C)** Wound-healing assay in TPC1 cells. The inhibitory effects on cell migration of TNRC6C-AS1 knockdown was attenuated by co-transfecting with TNRC6C-siRNA2. The promotive effect on cell migration of TNRC6C knockdown was reduced by by co-transfecting with TNRC6C-AS1-siRNA2 [representative images (left) and quantitative analysis (right)]. **(D)** Transwell assay in TPC1 cells. The inhibitory effects on cell invasion of TNRC6C-AS1 knockdown was attenuated by co-transfecting with TNRC6C-siRNA2. The promotive effect on cell invasion of TNRC6C knockdown was reduced by by co-transfecting with TNRC6C-AS1-siRNA2 [representative images (left) and quantitative analysis (right)]. *Compared with control siRNA transfecting TPC1 cells, #compared with TNRC6C-siRNA2 and TNRC6C-AS1-siRNA2 co-transfecting TPC1 cells. *or #*P* < 0.05;**or ##*P* < 0.01;***or ###*P* < 0.001.

To gain further insights into the significance of TNRC6C-AS1 –TNRC6C signaling in PTC, we examined the functional effects of TNRC6C and TNRC6C-AS1 down-regulated TPC1 cells by co-transfecting with two siRNAs. The CCK-8 assay showed that the inhibitory effect of TNRC6C-AS1 knockdown on cell proliferation was attenuated when the expression of TNRC6C was suppressed by co-transfecting with TNRC6C-siRNA2 for 48 h (Figure 5A). Similarly, promotive effect of TNRC6C knockdown on cell proliferation was reduced by co-transfecting with TNRC6C-AS1-siRNA2 for 48 h (Figure 5A). The flow cytometry assay revealed that the promotive effect of TNRC6C-AS1 knockdown on cell apoptosis was decreased by co-transfecting with TNRC6C-siRNA2 for 48 h (Figure 5B). Similarly, the inhibotory effect of TNRC6C knockdown on cell apoptosis was reduced by co-transfecting with TNRC6C-AS1-siRNA2 for 48 h (Figure 5B). The wound-healing assay and transwell assay revealed that the inhibitory effects on cell migration and invasion of TNRC6C-AS1 knockdown was attenuated by co-transfecting with TNRC6C-siRNA2 (Figures 5C,D). Similarly, the promotive effect on cell migration and invasion of TNRC6C knockdown was reduced by co-transfecting with TNRC6C-AS1-siRNA2 (Figures 5C,D). Taken together, we considered that TNRC6C is a functional target of TNRC6C-AS1 and TNRC6C-AS1 - TNRC6C signaling regulates the behavior of TPC1.

### Downregulation of TNRC6C-AS1 or overexpression of TNRC6C promotes iodine uptake of TPC1 cells

**Figure 6:**
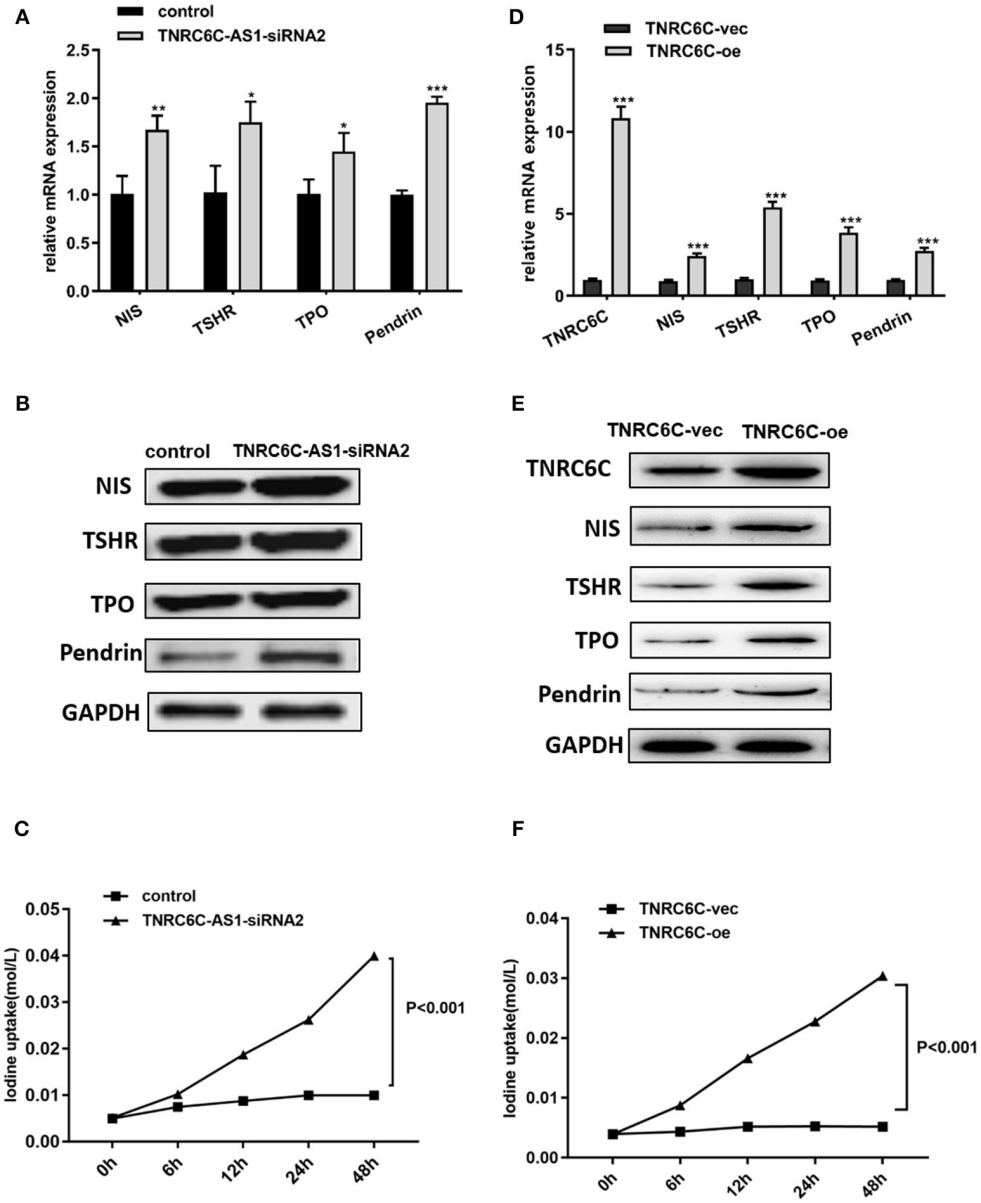
Downregulation of TNRC6C-AS1 or overexpression of TNRC6C promotes iodine uptake of TPC1 cells. **(A)** Downregulation of TNRC6C-AS1 by transfecting of siRNA-TNRC6C-AS1 for 48 h caused significantly increased mRNA expression of NIS, TPO, TSHR and Pendrin in TPC1 cells compared to control cells. **(B)** Downregulation of TNRC6C-AS1 by transfecting of siRNA-TNRC6C-AS1 for 48 h showed significant increase of protein levels of NIS, TPO, TSHR and Pendrin in TPC1 cells (GAPDH was used as the internal control). **(C)** Iodine uptake assay showed that iodide accumulation in TPC1 cells was significantly increased after TNRC6C-AS1 knockdown compared to control cells at 6, 12, 24, and 48 h. **(D)** Overexpression of TNRC6C by transfecting of TNRC6C overexpression plasmid for 48 h caused significantly increased mRNA expression of NIS, TPO, TSHR and Pendrin in TPC1 cells compared to control cells. **(E)** Overexpression of TNRC6C by transfecting of TNRC6C overexpression plasmid for 48 h showed significant increase of protein levels of NIS, TPO, TSHR, and Pendrin in TPC1 cells compared to control cells. (GAPDH was used as the internal control). **(F)** Iodine uptake assay showed that iodine accumulation in TPC1 cells was significantly increased after TNRC6C overexpression compared to control cells at 6, 12, 24, and 48 h. vec, empty vector; oe, overexpression vector; *P < 0.05; **P < 0.01; ***P < 0.001.

To explore the effect of TNRC6C-AS1 on iodine uptake, we knocked down TNRC6C-AS1 and measured the expression of iodine metabolism genes in TPC1 cells, including NIS, TPO, TSHR and Pendrin. The results showed that the mRNA and protein levels of NIS, TPO, TSHR and Pendrin were significantly increased in TNRC6C-AS1 knockdown TPC1 cells (Figures 6A,B). Consistently, iodide uptake assay showed that iodide accumulation was significantly increased after TNRC6C-AS1 knockdown at 6, 12, 24 and 48 h (Figure 6C). We also investigated the effect of TNRC6C on iodine uptake. We found that relative to empty vector transfected TPC1 cells, the expression of iodine metabolism genes and iodide accumulation were significantly increased in TNRC6C over-expressed TPC1 cells (Figures 6D–F).

### TNRC6C-AS1 expression is inversely correlated with TNRC6C expression in PTC

**Figure 7:**
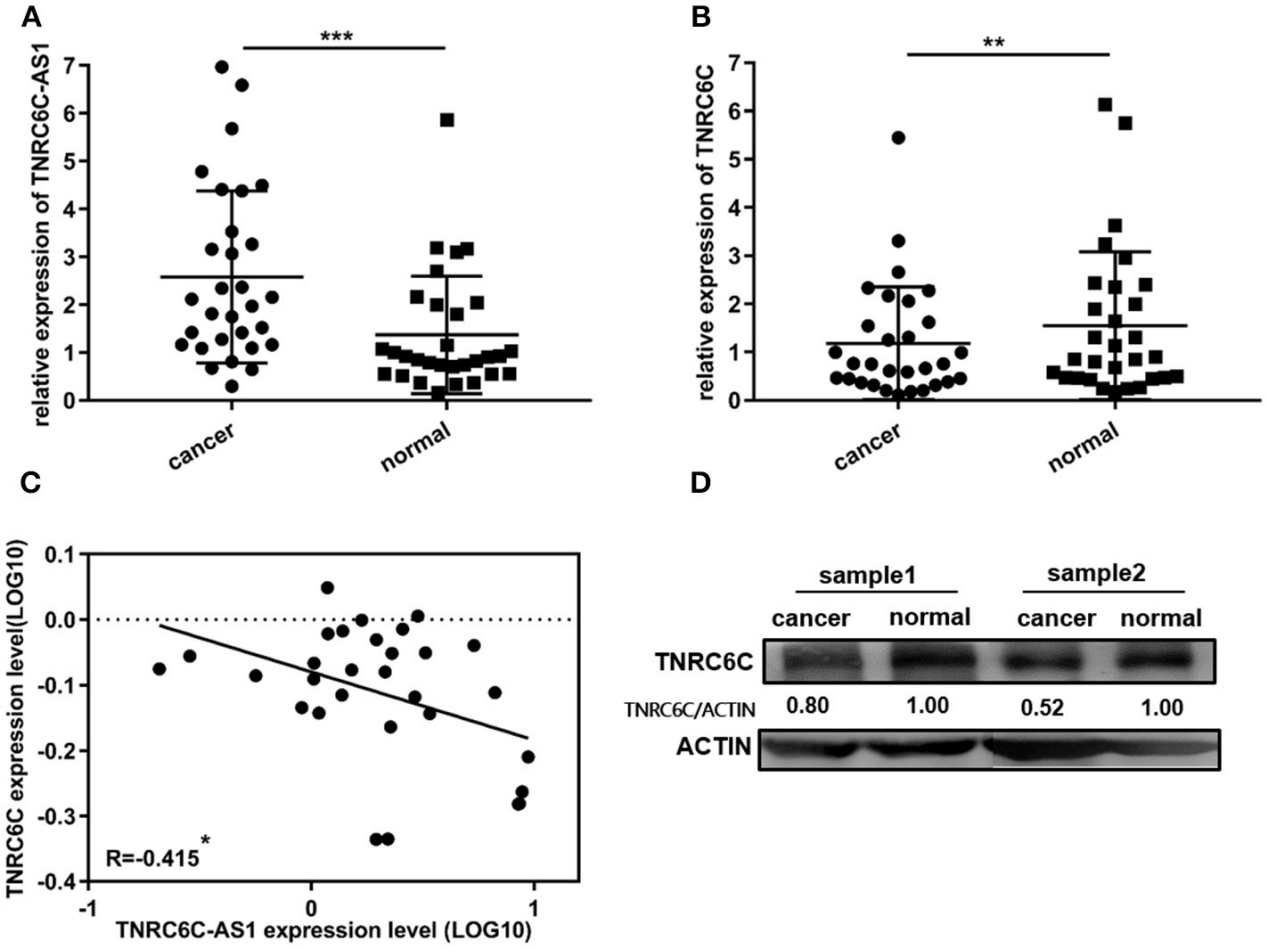
TNRC6C-AS1 and TNRC6C expression in PTC tissues and adjacent noncancerous tissues. **(A)**TNRC6C-AS1 RNA expression was significantly elevated in PTC tissues relative to the adjacent noncancerous tissues (*n* = 30). **(B)** TNRC6C mRNA expression in PTC tissues were significantly lower than the expression in adjacent normal tissues (*n* = 30). **(C)** Expression levels of TNRC6C-AS1 and TNRC6C mRNA were negatively correlated (*n* = 30) (*r* = −0.415, *P* < 0.05). **(D)**TNRC6C protein expression in PTC tissues were significantly lower than the expression in adjacent normal tissues (ACTIN was used as the internal control). **P < 0.01; ***P < 0.001.

30 pairs of PTC tissues and adjacent noncancerous tissues were used to confirm the expression levels of TNRC6C-AS1 and TNRC6C. TNRC6C-AS1 mRNA expression was significantly elevated in PTC tissues relative to the adjacent noncancerous tissues (Figure 7A). On the contrary, PTC tissues expressed significantly lower levels of TNRC6C mRNA and protein compared with adjacent normal tissues (Figures 7B,D). Next, we performed a correlation analysis of TNRC6C-AS1 and TNRC6C expression levels in the same PTC tissues. The result showed that the expression levels of TNRC6C-AS1 and TNRC6C mRNA were negatively correlated (*R* = −0.415, *P* < 0.05, Figure 7C), supporting the repressive effect of TNRC6C-AS1 on TNRC6C.

## Discussion

Actions and mechanisms of lncRNAs in PTC remain largely unknown. Herein, we first demonstrated an oncogenic role of a long non-coding antisense RNA TNRC6C-AS1 in tumorigenesis and invasiveness of PTC. We found that TNRC6C-AS1 was upregulated in PTC tissues, and it augmented proliferation, migration and invasion of TPC1 cells. We also found that the expression of TNRC6C-AS1 was inversely associated with its coding sense partner, TNRC6C mRNA, in PTC tissues. Downregulation of TNRC6C-AS1 expression resulted in increased TNRC6C mRNA and protein accumulation in TPC1 cells.

In general, NATs can be classified into cis-antisense transcripts which are encoded at the same genetic location but on the opposite strand to the RNAs that they act on, and trans-antisense transcripts which are encoded at a chromosomal location different from the RNAs that they act on Wahlestedt (25). Overlaps of cis sense/antisense pairs can target different portions of the corresponding transcriptional unit, giving rise to the following types of sense/antisense pairs: head-to-head or divergent (5′-regions overlap), tail-to-tail or convergent (3′-regions overlap), embedded (one transcript is fully contained within the other), or intronic pairs (10, 26). It was suggested that two types of regulation may occur between an antisense transcript and its cognate sense gene: discordant and concordant (10, 25, 26). As a result, TNRC6C-AS1 and TNRC6C are “tail to tail” cis sense/antisense pairs and are expressed in a discordant manner. Although no functional relevance has been contributed to the target positions of the antisense RNA, it has been shown that the “tail to tail” configuration was overrepresented in the discordant expression sense/antisense pairs (26).

Based on the observations that TNRC6C-AS1 regulated TNRC6C at both the mRNA and protein levels, we examined the role of TNRC6C-AS1–TNRC6C axis on the behavior of TPC1 cells to establish that TNRC6C was a functional target of TNRC6C-AS1. It was shown that downregulation of TNRC6C promoted proliferation, migration and invasion of TPC1 cells, while TNRC6C-AS1 knockdown had tumor-suppressive effects. The inhibitory effect of TNRC6C-AS1 knockdown on cell growth, migration and invasion was reduced when the expression of TNRC6C was suppressed at the same time. These experiments indicated that TNRC6C-AS1–TNRC6C axis has important effect on behavior of TPC1 cells, and TNRC6C-AS1 promote tumorigenesis and invasiveness of PTC through down-regulating the expression of the tumor-suppressive gene TNRC6C.

The cis-regulation of NATs can lead to activation or silencing of the corresponding sense mRNAs. Multiple mechanisms have been proposed, including transcriptional activation, silencing, mRNA stabilization, alternative splicing, or post-translational regulation (12, 27–29). As the overlapping region between TNRC6C-AS1 and TNRC6C positions to the 3′UTR region of the TNRC6C gene, we performed a luciferase reporter assay to find out if there was a direct interaction between TNRC6C-AS1 and TNRC6C 3′UTR. Interestingly, we found that TNRC6C-AS1 overexpression decreased relative luciferase activity in TPC1 cells. This finding suggests that TNRC6C-AS1 does not directly interact with TNRC6C 3′UTR, but it regulates the expression of TNRC6C through other mechanisms. The underlying mechanism of the cis-regulation of TNRC6C-AS1 on TNRC6C should be explored in further studies.

It is well known that dysregulation of miRNA is closely associated with cancer development, in which miRNAs act as either tumor suppressors or tumor promoters by regulating proliferation, apoptosis, invasion and metastasis (30, 31). Evidences have shown that TNRC6C is a miRNA regulation-related gene and is the key component for miRNA-mediated mRNA repression (14–17). The expression of TNRC6 proteins was down-regulated in gastric, colorectal and non-small cell lung cancers in previous studies (18, 19) as well as in PTC in our study, suggesting TNRC6C may be involved in the repression of some oncogenes through miRNA-dependent gene silencing. We considered that down-regulation of TNRC6C expression may lead to increased expression of some miRNA-regulated oncogenes. In fact, we have predicted some oncogenes which are involved in the progression of PTC, and one of the potential target genes is Dipeptidyl Peptidase IV (DPP4). Recently, Lee et al. have confirmed that DPP4 expression was significantly higher in PTC tissues than normal adjacent tissues, and increased DPP4 expression was associated with cellular invasion and more aggressive disease in PTC. Besides, high expression of DPP4 was associated with some clinicopathological factors, including larger sizes of tumor, lymphatic metastasis, higher TNM stages, higher recurrence rate and unfavorable histological type (32). We investigated the TNRC6C and DPP4 mRNA expression in 501 PTC tissues by using The Cancer Genome Atlas (TCGA) database. There was a significant inverse correlation between TNRC6C and DPP4 mRNA expression in PTC tissue samples (*r* = −0.27, *P* < 0.0001). Therefore, DPP4 may be the potential target gene regulated by the TNRC6C-AS1 - TNRC6C axis. On the basis of these observations, next studies are needed to validate the target oncogenes indicated.

Differentiated thyroid cells express a number of differentiation markers such as TSHR, NIS, Pendrin, and TPO, which are responsible for iodine uptake, synthesis of thyroid hormones as well as the differentiated thyroid phenotype (33). A loss of or decrease in specific markers of functional differentiation in thyroid carcinomas is associated with these proteins involved in thyroid hormone production, thus dedifferentiated thyroid carcinomas lose or have defect in their capacity of iodide transportation and iodination of thyroglobulin (33). In this study, we also found that the expression of iodine metabolism genes, including NIS, TPO, TSHR, and Pendrin, were upregulated by knocking down TNRC6C-AS1 or overexpressing TNRC6C. Consistently, the ability of iodine uptake of TPC1 cells was increased. These findings suggest that dysregulation of the TNRC6C-AS1–TNRC6C axis plays a role in the de-differention of PTC, and modulating this axis can promote the re-differention of PTC. Dedifferentiated thyroid carcinoma with the loss of radioiodine uptake is frequently observed in PTC patients under radioiodine therapy, indicating a poor prognosis (23). Modulating the TNRC6C-AS1–TNRC6C axis would contribute to the recovery of radioiodine-sensitivity in dedifferentiated PTC through positively regulating the iodine metabolism genes. Therefore, the TNRC6C-AS1–TNRC6C axis may serve as a potential treatment target to improve the efficacy of radioactive therapy of dedifferentiated PTC.

In summary, we identified a regulatory relationship between an antisense lncRNA TNRC6C-AS1 and its coding sense partner TNRC6C in PTC. We demonstrated that TNRC6C-AS1 promotes the progression of PTC and inhibits its ability of iodine accumulation by suppressing the expression of TNRC6C, and restoration of TNRC6C expression can reverse these effects. Our study suggests that TNRC6C-AS1–TNRC6C axis has a role in tumorigenesis, invasiveness, and iodine accumulation of PTC, and restoration of the TNRC6C-AS1–TNRC6C axis may be a new promising treatment for PTC.

## Author contributions

DM and TZ contributed equally to this work. YL conceived and designed the study. DM and TZ performed the experiments and analyzed the data. JJ, ZA, and WZ contributed to the experiments and data analysis. DM, TZ, and YL wrote and revised the paper.

### Conflict of interest statement

The authors declare that the research was conducted in the absence of any commercial or financial relationships that could be construed as a potential conflict of interest.
