# Supplementary material for: Long Non-coding Antisense RNA TNRC6C-AS1 Is Activated in Papillary Thyroid Cancer and Promotes Cancer Progression by Suppressing TNRC6C Expression
Source: Front Endocrinol (Lausanne). 2018 Jul 9;9:360. doi: 10.3389/fendo.2018.00360 (PMC6046411; doi:10.3389/fendo.2018.00360)
Supplement: Supplementary file 1 [file Table_1.DOCX]

**Supplementary Tabel S1 Primer and siRNA sequences for this study**

| **Gene** | **Forward Primer** | **Reverse Primer** |
| --- | --- | --- |
|  | **(sense)** | **(anti-sense)** |
| **TNRC6C-AS1** | GTGCGAGTGCTGCTGGGTGAAG | GAGGGACCGTGATGGTTTGGAG |
| **TNRC6C** | CGGCATCTTCTGGAACTAC | CCCTTTATCTCGCCTTCG |
| **NIS** | TAAGTGGCTTCTGGGTTGTCC | ATAGCGGCTCCTCGGGTCA |
| **TSHR** | TCACCAGCCACTACAAAC | AGGTCTACAGAGGCGATG |
| **TPO** | ACACTTGCCTGGCGAACA | GACTGAAGCCGTCCTCATAGA |
| **Pendrin** | ATCCCAACCAAGGAAATAGA | CTTTGACAATCACCCGCAGT |
| **GAPDH** | ACAACTTTGGTATCGTGGAAG | GCCATCACGCCACAGTTTC |
| **β-actin** | TCACCCACACTGTGCCCATCTACGA | CAGCGGAACCGCTCATTGCCAATGG |
| **TNRC6C-AS1-siRNA1** | CCCAAGAGUUUCGGUUUAUTT | AUAAACCGAAACUCUUGGGTT |
| **TNRC6C-AS1-siRNA2** | CCCGAGAGAUUCUGAUUGATT | UCAAUCAGAAUCUCUCGGGTT |
| **TNRC6C-AS1-siRNA3** | GGCCGGAUGUGAAUUAUCUTT | AGAUAAUUCACAUCCGGCCTT |
| **TNRC6C-siRNA1** | GCUGGGAUAAAGUGAUAAUUU | UUGACCCUAUUUCACUAUUAU |
| **TNRC6C-siRNA2** | CUCCAAUGCUGGCAUUAAUUU | UUGGCGAGAUAAAGGGAUUAU |
| **TNRC6C-siRNA3** | GCGAGAUAAAGGGAUUAUAUU | UUGCGGGAAGGAAAUCGAUUA |
